# Membrane Activity of Melittin and Magainin-I at Low Peptide-to-Lipid Ratio: Different Types of Pores and Translocation Mechanisms

**DOI:** 10.3390/biom14091118

**Published:** 2024-09-04

**Authors:** Marta V. Volovik, Oleg V. Batishchev

**Affiliations:** Laboratory of Bioelectrochemistry, A.N. Frumkin Institute of Physical Chemistry and Electrochemistry, Russian Academy of Sciences, 31/4 Leninskiy Prospekt, 119071 Moscow, Russia; marta.volovik@phystech.edu

**Keywords:** antimicrobial peptides, melittin, magainin, bilayer lipid membranes, membrane activity, membrane potentials, pore formation, patch-clamp, peptide–lipid interactions, adsorption

## Abstract

Antimicrobial peptides (AMPs) are believed to be a prominent alternative to the common antibiotics. However, despite decades of research, there are still no good clinical examples of peptide-based antimicrobial drugs for system application. The main reasons are loss of activity in the human body, cytotoxicity, and low selectivity. To overcome these challenges, a well-established structure–function relationship for AMPs is critical. In the present study, we focused on the well-known examples of melittin and magainin to investigate in detail the initial stages of AMP interaction with lipid membranes at low peptide-to-lipid ratio. By combining the patch-clamp technique with the bioelectrochemical method of intramembrane field compensation, we showed that these peptides interact with the membrane in different ways: melittin inserts deeper into the lipid bilayer than magainin. This difference led to diversity in pore formation. While magainin, after a threshold concentration, formed the well-known toroidal pores, allowing the translocation of the peptide through the membrane, melittin probably induced predominantly pure lipidic pores with a very low rate of peptide translocation. Thus, our results shed light on the early stages of peptide–membrane interactions and suggest new insights into the structure–function relationship of AMPs based on the depth of their membrane insertion.

## 1. Introduction

Increasing resistance of pathogens to traditional antibiotics is a widespread threat to global health [1,2,3,4,5,6]. Antibiotics currently used for treatment are becoming less effective, and the number of new antibiotics has continued to decline over the past decades [1,7]. An alternative strategy to combat harmful microorganisms is the introduction of antimicrobial peptides (AMPs) into medical therapy. AMPs pretend to be a new generation of antibiotics. Although they have a broad spectrum of activity against multidrug-resistant microbes [8,9,10,11] and are known to be expressed by the immune system of a large variety of species [12], clinical trials face a number of difficulties in using AMPs as medical drugs, such as high cytotoxicity to eukaryotic cells, enzymatic degradation in the human body, and low selectivity [13,14]. Understanding the relationship between the structure and function of AMPs would overcome these difficulties. Currently, it is known that AMPs act directly on bacterial membranes, disrupting them and making it more difficult for pathogens to develop resistance [1,5]. Typical AMPs are amphipathic molecules that bear a net positive charge and comprise up to 50 amino acid residues [15]. More than 50% of AMP residues are hydrophobic [16], which drives peptide insertion into the lipid membrane. Upon adsorption onto the membrane, AMPs acquire predominantly α-helical, β-hairpin, or β-sheet secondary structure [17]. The α-helical peptides are the most extensively studied examples of AMPs. They are known to initially adsorb approximately parallel to the membrane plane in the so-called S-configuration [18,19,20]. Reaching a threshold concentration at the membrane surface causes the rearrangement of the peptides from the S-configuration to the I-configuration, which is described as insertion of the peptide deeply into the lipid bilayer perpendicular to the membrane plane [18,19,20,21]. This state is associated with the onset of pore formation and subsequent membrane disruption. Several major models have been proposed for the AMP membrane activity: toroidal pore, barrel-stave pore, or detergent-like action, known as the carpet model [18,22,23]. According to the carpet model [24], peptides solubilize the membrane, eventually causing the micelle formation. The barrel-stave model [25] is associated with the pore being completely lined by peptides tightly assembled at the pore edge, whereas the walls of the toroidal pore are assumed to consist of both peptides and lipids [22]. Nevertheless, the precise transition pathways between the S-configuration and the I-configuration remain unclear.

In contrast to the classical ionic channels, AMPs form pores varying in size and structure with a fluctuating lifetime. According to the studies of the membrane conductance induced by the AMP, the electrical signals corresponding to pore formation cannot be described by a monotonic increase in conductance [26,27,28]. Instead, the changes in the membrane conductance can be classified into three main types: spike, multi-level, and square-top signals [29]. The spike signal is the shortest instantaneous fluctuation. The multi-level signal is a set of conductance jumps that are much longer than the spike signals. The square-top signal reflects some stable, long-living structure and is characterized by a step-like time-dependence of the conductance. Some authors associate each type of signal with pore structure. For example, the formation of barrel-stave or toroidal pores is assumed to result in square-top or multi-level signals, respectively [26]. However, while the antimicrobial peptide melittin forms toroidal pores, it induces either a large variety of square-top conductance signals [27]. As for the spike signal, it is assumed to reflect the minor perturbations of the membrane that some authors relate to the peptide translocation across the membrane. However, peptide translocation can occur without the formation of pores, which is leaky for water-soluble fluorescent dyes [30]. On the other hand, two AMP molecules can form a purely lipidic pore between them [31]. Thus, the nature of the spike signals is much more complex and may even reflect the formation of lipidic pores.

Although each type of electrical signal varies considerably in its shape, amplitude, and duration, all of them are finite, which is apparently caused by pore closure over time. This raises the question of the mechanisms of pore formation, evolution, and inevitable disappearance. The time-limited permeabilization of the membrane, even at high concentrations of the peptides, has also been observed for AMP in the experiments on the leakage of water-soluble fluorescent dye from the vesicles [32,33], and can also be related to the pore closure. In the work [34], the authors have suggested that the peptide redistribution between two leaflets of the membrane due to the AMP translocation across the membrane upon pore formation causes the pore closure. In fact, peptide translocation has been demonstrated in a number of studies. Matsuzaki et al. have shown using FRET that the fluorescently labeled peptide, which is added to one side of the membrane, translocated across the lipid bilayer [34,35]. However, the translocation can take place via the lipidic pores, which continuously connect two membrane leaflets, allowing them to exchange lipids and peptides. In the study of the pore formation induced by magainin-2 [36], the authors also note that peptide translocation along the wall of the pores may facilitate the flip-flop of the lipids. Surprisingly, peptide translocation across the membrane of the vesicles can occur even without any dye leakage, as has also been observed using confocal fluorescence microscopy [30]. The ability of AMP to pass through the membrane without any detectable pore formation has also been shown in the work [37].

Since antimicrobial peptides are charged molecules, the charge of lipids and transmembrane potential differences should influence AMP membrane binding and subsequent pore formation. The binding constant of the antimicrobial peptide magainin-2 to the outer leaflet of the GUV membrane has been shown to increase with the increasing transmembrane potential difference [38]. Furthermore, the negative voltage applied to the membrane is known to facilitate peptide adsorption, insertion, and translocation across the membrane [34,39,40].

Melittin and magainin-I (hereinafter referred to as magainin) are the two well-known and representative examples of the antimicrobial peptides that translocate across the membrane and form lipid–peptide pores conforming to the toroidal pore model. The former peptide is part of the venom of the European honeybee *Apis mellifera*, while the latter one has been isolated from the African frog skin *Xenopus Laevis* [9,41]. Melittin and magainin carry positive charges of +6*e* [23,42] and +5*e* [22,43,44] and consist of 23 and 26 amino acid residues, respectively. They both fold into an α-helix at the membrane surface and are about 3.5 nm long. Meanwhile, the differences between magainin and melittin have already been noted in early studies. Melittin is known to predominantly adsorb onto the membrane in the form of a monomer [23,45], while at higher surface concentrations on the membrane it is supposed to self-assemble into aggregates with subsequent peptide reorientation and insertion into the lipid bilayer. The formation of clusters of melittin has been shown by several molecular dynamics (MD) simulations [44,46]. According to the MD simulations [44], melittin can form 4–6 peptide assemblies in the pore region, and the presence of the proline residue enables it to form U-shaped conformation in the membrane, promoting easier peptide incorporation into the lipid bilayer [23]. In contrast to melittin, magainin binds to the membrane surface as a dimer, as follows from the NMR and fluorescence studies [47,48]. Presumably, due to the lower charge, magainin tends to aggregate into larger clusters in the pore region, which has been confirmed by the MD simulation experiments [44]. Nevertheless, additional simulations with melittin demonstrated that the threshold concentration of the pore formation and the peptide-to-lipid (P/L) ratio are not the key factors. Instead, the local concentration and distribution of the peptide on the membrane surface play a primary role in the AMP reorientation and insertion into the lipid bilayer [23]. In the work [49], the authors using coarse-grained MD simulations suggested that at high peptide-to-lipid ratios (P/L = 1/30), the membrane stress induced by magainin is greater than that of melittin. Thus, it may lead to different modes of pore formation to release the membrane stress.

In our study, we investigated the interaction of the antimicrobial peptides melittin and magainin with the planar lipid bilayers at low peptide concentrations using a unique bioelectrochemical method of intramembrane field compensation (IFC) [50]. These two peptides have been chosen as representative examples of the most widely studied pore-forming α-helical antimicrobial peptides. However, even for those peptides, the whole trajectory of the adsorption-pore formation-possible translocation and pore closure is still unknown. IFC allows to monitor both adsorption and translocation of charged molecules at lipid membranes. We studied the initial stages of the adsorption of charged peptide molecules onto the anionic lipid membranes at low AMP concentrations and detected the peptide translocation across the membrane by measuring the change in the boundary potential difference across the membrane. To characterize pore formation mechanisms in detail, we performed electrophysiological experiments using the patch-clamp technique. This allowed us to determine the threshold concentrations of the peptides required to induce pore formation. Comparing the results obtained from patch-clamp and IFC experiments, we made conclusions on the pore formation and translocation mechanisms for melittin and magainin.

## 2. Materials and Methods

### 2.1. Materials

The lipids 1,2-dioleoyl-*sn*-glycero-3-phosphocholine (DOPC), 1,2-dioleoyl-*sn-*glycero-3-phospho-rac-(1-glycerol) sodium salt (DOPG), 1,2-dioleoyl-*sn*-glycero-3-phosphoethanolamine (DOPE) were from Avanti Polar Lipids (Alabaster, AL, USA). Lipid mixtures were prepared using chloroform, decane, squalane, octane (all from Sigma Aldrich, Saint-Louis, MO, USA, purity >99%). Buffer solutions were prepared using KCl (Sigma-Aldrich, Saint-Louis, MO, USA) and HEPES (Helicon, Moscow, Russia) in double-distilled water (MilliPore, Direct-Q 3UV system, Burlington, MA, USA). Experiments were performed with melittin and magainin-1 (both purchased from Sigma-Aldrich, Saint-Louis, MO, USA, purity >97%, HPLC) without further purification.

### 2.2. ζ-Potential Measurements

For ζ-potential measurements in a suspension of liposomes, we produced sonicated lipid vesicles. Lipid mixture DOPC:DOPE:DOPG in a molar ratio of 60:20:20% was dissolved in chloroform to the concentration of 10 mg/mL and then evaporated in a round-bottomed glass flask in rotary evaporator (Büchi Labortechnik, Flawil, Switzerland) for 40 min at 40 mbar pressure. The dried lipid film was then hydrated with the buffer solution of low ionic strength (10 mM KCl, 5 mM HEPES, 0.1 mM EDTA, pH 7.5) and sonicated for 30 min to obtain the liposomes. The final lipid concentration in the buffer solution was 1 mg/mL.

Measurements of the ζ-potential of the liposomes were performed to obtain the surface potential value [51]. In the experiments, we recorded electrophoretic mobility of liposomes utilizing the dynamic light scattering technique on a Zetasizer II device (Malvern Instruments, Malvern, Worcestershire, UK) with the PhotoCor SP correlator (PhotoCor, Moscow, Russia). All measurements were made in low ionic strength conditions (10 mM KCl, 5 mM HEPES, 0.1 mM EDTA, pH 7.5) in double-distilled water.

### 2.3. Patch-Clamp Experiments

Planar bilayer lipid membranes (BLMs) were formed on the 100 µm x 100 µm copper mesh using the Muller-Rudin technique [52]. To form the BLMs, the lipids were mixed in chloroform at a concentration of 10 mg/mL in a 1.5 mL Eppendorf vial, and the chloroform was evaporated under an argon stream. The obtained lipid film of DOPC:DOPE:DOPG in a molar ratio of 60:20:20% was then dissolved in a mixture of octane and decane (1:1) in a concentration of 10 mg/mL or in squalane in a concentration of 20 mg/mL. The copper mesh was then pretreated with 0.6 µL of the lipid mixture in octane and decane and dried until a meniscus was formed. The squalane-lipid mixture was applied to the mesh placed in the working buffer solution (100 mM KCl, 10 mM HEPES, pH 7.5) in a Petri dish, and the lipid membranes were formed spontaneously.

To study the ionic conductance of the membranes under the action of melittin and magainin, we used the patch-clamp technique. The measuring electrode was placed in the micropipette filled with the peptide solution in the working buffer, and the ground electrode was placed in the buffer solution in the Petri dish. After formation of a tight contact between the micropipette and the planar lipid bilayer, a constant voltage of +100 mV was applied to the membrane using a patch-clamp amplifier (HEKA EPC-8, Lambrecht, Germany), and the conductance of the membrane was monitored. Electrical measurements were performed with pore-forming concentrations of the peptides: 140 nM melittin and 160 nM magainin. Additionally, measurements were performed using a concentration that was twofold lower: 70 nM melittin and 80 nM magainin in the micropipette. For each concentration, 5 experiments of ten minutes each were performed.

To analyze the obtained results in detail, we classified all electrical signals into three types: spike, multi-level, and square-top signals, as suggested in the work [29]. Then, we calculated the number of each conducting event (spike, multi-level, or square-top signal) for each minute of the recording (total duration 10 min) and divided them by the total number of trials (n = 5). The results of the calculations were presented as the relative occurrence of conducting events.

### 2.4. Intramembrane Field Compensation Method

Planar BLMs were formed at the aperture with a diameter of 1 mm in a Teflon partition separating two Teflon chambers of 2 mL volume each, using the Muller-Rudin technique [52]. The chambers were filled with a working buffer solution of high ionic strength (100 mM KCl, 10 mM HEPES, pH 7.5) or low ionic strength (10 mM KCl, 5 mM HEPES, pH 7.5). Both compartments were stirred continuously with magnetic stirrers. To form BLMs, the lipids were mixed in chloroform at a concentration of 10 mg/mL in a 1.5 mL Eppendorf vial, and the chloroform was dried under an argon stream. The resulting lipid film of DOPC:DOPE:DOPG in a molar ratio of 60:20:20% was then dissolved in decane at a concentration of 20 mg/mL. For electrical measurements, two Ag/AgCl electrodes were placed in the agar bridges filled with the buffer solution in the Teflon chambers. The bridges were made of plastic micropipette tips containing agar and 100 mM KCl. Melittin or magainin was then added to the buffer solution in one of the Teflon chambers. Stock solutions of melittin and magainin were dissolved in double-distilled water to a concentration of 20 μg/mL of the peptide. The volume of each addition of peptide solution to the Teflon chamber was 20 μL.

**Figure 1:**
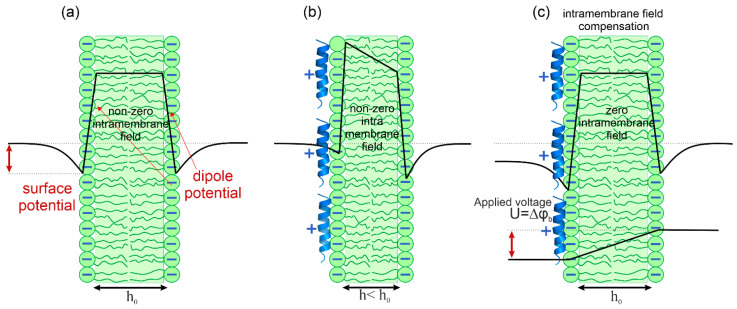
Scheme of the method of intramembrane field compensation. The anionic membrane without peptide adsorption when the intramembrane field is zero (**a**). The boundary potential is a sum of the surface potential measured in the diffuse part of the electric double layer and the dipole potential resulting from the mutual orientation of the water dipoles near the membrane and the dipoles of the polar heads of the lipids. The thickness of the lipid bilayer is h_0_. Adsorption of charged AMP onto the anionic membrane induces intramembrane field, which causes a decrease in membrane thickness (h < h_0_) and an increase in membrane capacitance due to electrostriction (**b**). Application of the external voltage (U = Δφ_b_) compensates the intramembrane field (**c**). The thickness of the lipid bilayer is h_0_ again.

Peptide adsorption was studied using the IFC method [50,53,54]. In this method, the lipid membrane is considered a planar elastic capacitor, the thickness of which decreases (and capacitance increases) with increasing transmembrane voltage due to electrostriction (see Figure 1). The boundary potential at one side of the membrane reflects the potential difference between a point in the bulk water solution far from the membrane and a point at the boundary between polar and nonpolar parts of lipid monolayer. Therefore, the boundary potential is a sum of the surface potential measured in the diffuse part of the electric double layer and the dipole potential resulting from the mutual orientation of the water dipoles near the membrane and the dipoles of the polar heads of the lipids. The minimal membrane capacitance corresponds to a zero difference of the membrane boundary potentials across the membrane (Δφ_b_). The one-sided adsorption of charged molecules leads to a change in the Δφ_b_ value across the membrane and thus to the appearance of the intramembrane electric field. By applying an external DC field (U), it is possible to make this field equal to zero when U = Δφ_b_ (Figure 1c). The self-made software automatically attenuates the amplitude of the DC voltage to compensate for the intramembrane field; the negative feedback loop sets it equal to the boundary potential difference. Therefore, by recording the value of U, one can monitor the kinetics of the change in the value of Δφ_b_.

In many studies, IFC method has been applied to study interaction of macromolecules, polypeptides, and proteins with lipid bilayer [53,55,56]. Thus, this method allows monitoring the adsorption and translocation kinetics of melittin and magainin at the BLM.

## 3. Results

### 3.1. ζ-Potential Measurements on Liposomes

In our study, we used the lipid composition of DOPC:DOPE:DOPG in a molar ratio of 60:20:20%, which contains both bacterial PE and PG lipids together with eukaryotic PC lipids. This lipid composition allows the preparation of both liposomes and stable planar bilayer lipid membranes and is frequently used in the studies of peptide–membrane interactions [57]. It is established that the presence of negatively charged lipids enhances membrane binding of antimicrobial peptides [58]. At the same time, they can interact with an uncharged lipid bilayer made of zwitterionic lipids [58]. From the point of view of the classical Gouy–Chapman–Stern (GCS) theory, the concentration of the charged molecules near the charged surface in solution should follow the Boltzmann distribution:

(1) $$C\left(0\right)=C_{0}\exp\left(-\frac{z_{i}F\varphi \left(0\right)}{RT}\right),$$

where *C*_0_ is the concentration of adsorbing molecules in solution, *z_i_* is the charge number of the *i*th molecule, *F* is the Faraday constant, *R* is the gas constant, *T* is the absolute temperature, and *φ*(0) is the surface potential at the membrane-water interface.

To assess the surface charge density, we measured ζ-potential in a suspension of liposomes of the used lipid composition of DOPC:DOPE:DOPG in a molar ratio of 60%:20%:20%. The obtained value was −39 ± 1 mV (SD, n = 5). In fact, the ζ-potential measured in the hydrodynamic slipping plane differs from the surface potential at the membrane surface. For lipid membranes, the slipping plane lies at the distance *x* = 0.2 nm from the membrane surface [59,60]. To calculate the value of *φ*(0), we should use the potential distribution predicted by the GCS theory:

(2) $$tanh\left(\frac{z_{i}F\varphi \left(x\right)}{4RT}\right)=\exp\left(-\kappa x\right)tanh\left(\frac{z_{i}F\varphi \left(0\right)}{4RT}\right),$$

where $\kappa =\sqrt{\frac{2z^{2}F^{2}C_{el}}{\varepsilon \varepsilon_{0}RT}}$ is the inverse Debye screening length, *z* and *C_el_* are the charge number and concentration of the binary electrolyte ions, and *ε* and *ε*_0_ are the dielectric constants in the solution and in vacuum, respectively. Thus, for the ζ-potential of −39 ± 1 mV, we obtained *φ*(0) = −42 ± 1 mV.

### 3.2. Patch-Clamp Experiments on Melittin and Magainin Pore Formation

**Figure 2:**
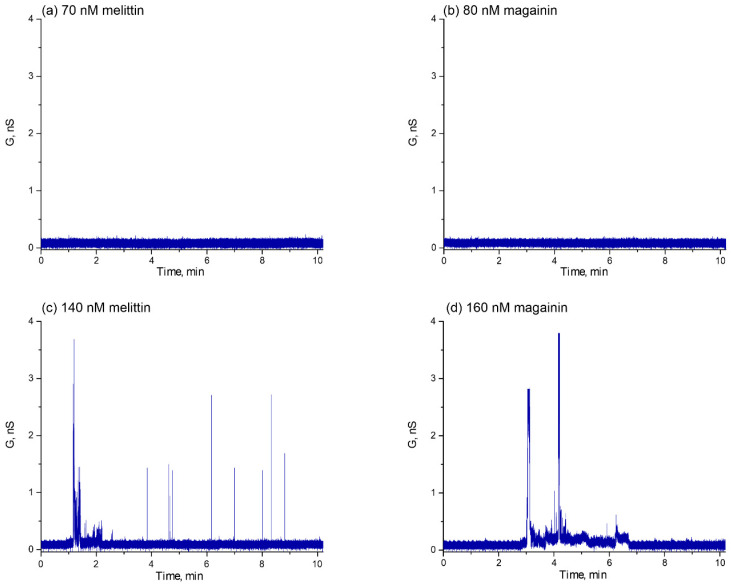
Typical kinetics of the membrane conductance under the action of 70 nM melittin (**a**); 80 nM magainin (**b**); 140 nM melittin (**c**); 160 nM magainin (**d**).

To analyze pore formation, pore structure, and peptide translocation through the pores, we performed electrophysiological experiments using the patch-clamp technique on planar lipid bilayers. The micropipette was filled with the peptide solution, and after formation of a tight contact between the lipid bilayer and the micropipette, the electric current through the membrane was recorded. Typically, antimicrobial peptides form pores in the P/L range of 1/25–1/100 [22,61]. The intrinsic binding constant for melittin is (4.5 ± 0.6) × 10^4^ M^−1^, and its charge number *z_i_* = 1.9 [62]. Taking into account *φ*(0) = −42 ± 1 mV for our lipid composition, the reported projection area of the peptide of 150 Å^2^ and the area per lipid of 68 Å^2^ [62], one can estimate that for melittin the value of P/L = 1/100 corresponds to the bulk concentration of approximately 140 nM. For magainin, the binding constant has been estimated as 55 ± 5 M^−1^ [63,64], while its charge number should be higher, around 3.7 [63,64]. Magainin forms dimers at the membrane surface, and its pore-forming concentration corresponds to the P/L = 1/60 [63,64,65]. For *φ*(0) = −42 ± 1 mV, the bulk concentration of magainin is about 160 nM. To independently verify these estimations, we started our patch-clamp experiment for twofold lower concentrations of 70 nM melittin and 80 nM magainin. Typical kinetics of the obtained membrane conductance are presented in Figure 2a,b. As expected, we did not detect any changes in the membrane conductance for these peptide concentrations. For 140 nM melittin and 160 nM magainin, these peptides induced a broad spectrum of membrane electrical activity. Typical kinetics of the membrane conductance are shown in Figure 2c,d.

**Figure 3:**
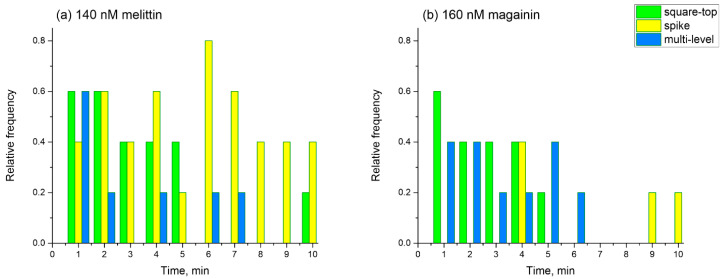
Histograms of the relative frequency of occurrence of spike, multi-level, square-top signals during 10 min of recording obtained from 5 experiments for 140 nM of melittin (**a**) and 160 nM of magainin (**b**).

To compare the conductance traces for melittin and magainin in detail, we classified all electrical signals into three types: spike, multi-level, and square-top signals. This classification was based on the analysis of the typical conductance activity of different channels observed in voltage-clamp experiments [29]. The distribution of each type of signal over the course of the experiment (10 min) for melittin and magainin is presented in Figure 3 as the histograms of the relative frequency of occurrence of spike, multi-level, and square-top signals. Comparing melittin and magainin activity, it can be noted that melittin induced predominantly spike signals (Figure 3a), while magainin formed larger long-living pores, reflected in the appearance of square-top signals, and induced only a slight spike conductance (Figure 3b).

It should be noted that for both peptides, the relative frequency of conducting events decreased with time. This fact suggests a possible cessation of pore formation with time, which is believed to be related to the possible peptide translocation [34,35] followed by inhibition of pore formation. At the same time, we have previously demonstrated that two peptide molecules would be enough to form a lipidic pore in a membrane [66]. These pores could release the stress of the asymmetric membrane tension resulting from the one-sided adsorption of the peptides onto the membrane [67]. However, the patch-clamp technique does not allow to investigate in detail the peptide adsorption and translocation processes.

### 3.3. Intramembrane Field Compensation Measurements of the Melittin and Magainin Adsorption and Translocation across the Membrane

The IFC method allows the kinetics of adsorption of charged molecules added to one side of the BLM to be monitored by the change in the boundary potential difference across the membrane (Δφ_b_). If peptide molecules translocate through the membrane, this difference should decrease as the charged species would be less asymmetrically distributed between the membrane and the leaflets, thus diminishing the Δφ_b_.

**Figure 4:**
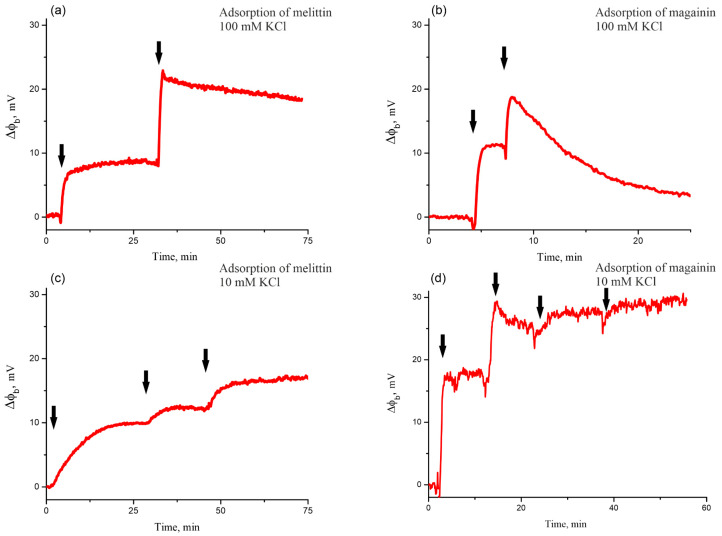
Typical kinetics of the change in the Δφ_b_ in the course of adsorption of melittin in the buffer solution with 100 mM KCl (**a**) and 10 mM KCl (**c**); in the course of adsorption of magainin in the buffer solution with 100 mM KCl (**b**) and 10 mM KCl (**d**). The black arrows indicate the addition of the peptide to the solution (70 nM melittin or 80 nM magainin).

The change in the Δφ_b_ was observed upon subsequent one-sided additions of melittin or magainin to the buffer solution in the vicinity of the membrane surface. Each addition step changed the peptide concentration in the buffer solution by 70 nM for melittin and 80 nM for magainin. First, we performed experiments in the same buffer conditions as for patch-clamp measurements (high ionic strength of 100 mM KCl). The typical kinetics of the change in the Δφ_b_ is shown in Figure 4a,b for melittin and magainin, respectively. For both peptides, the first and second additions led to almost equal change in the Δφ_b_. For melittin, we observed the change in the Δφ_b_ of 10 ± 3 mV (SD, n = 5) after the first addition and 8 ± 3 mV (SD, n = 4) after the second addition of the peptide, while in the case of magainin, the first addition of the peptide led to the change in the Δφ_b_ of 8 ± 3 mV (SD, n = 4) and the second addition resulted in a change in the Δφ_b_ of 7 ± 2 mV (SD, n = 4) (see Table 1). However, after the second addition of magainin, we observed a sharp decrease in the Δφ_b_ of about 15 mV, reaching almost the initial values of the Δφ_b_ in 10 min (Figure 4b). An exponential decay fit of the Δφ_b_ decrease resulted in a characteristic time of 9 ± 2 min (SD, n = 4) (Table 1). In contrast to magainin, we observed only a slight decrease in the Δφ_b_ for melittin after the second addition. The Δφ_b_ decreased by approximately 2 mV in 30 min, with a characteristic decay time of 70 ± 7 min (SD, n = 4) (Table 1).

To verify whether the ionic strength of the buffer solution influences the adsorption of the peptides in these concentrations, melittin or magainin was added to the BLM in a low-ionic strength buffer solution of 10 mM KCl. The typical kinetics of the change in the Δφ_b_ are shown in Figure 4c,d. For melittin, we observed a change in the Δφ_b_ of 9 ± 2 mV (SD, n = 4) after the first addition and 5 ± 2 mV (SD, n = 4) after the second addition of the peptide (see Table 1). For magainin, we observed a change in the Δφ_b_ of 16 ± 3 mV (SD, n = 4) after the first addition and of 11 ± 2 mV (SD, n = 4) after the second addition (Table 1). Noteworthy, no long-term decrease in the Δφ_b_, which can be approximated by an exponential decay fit, was observed for either melittin or magainin, even with subsequent additions.

## 4. Discussion

Almost since the invention of the first antibiotics, we have witnessed the first signs of their fading due to the emergence of drug-resistant bacteria [1]. Therefore, many researchers have proposed the idea of searching for the prominent antimicrobial drug among those invented by nature itself [1,5]. These substances are antimicrobial peptides produced by many organisms to fight bacteria. However, despite several decades of attempts to make a good peptide-based antibiotic, we are still limited to a few examples, none of which is suitable for system application due to high cytotoxicity to eukaryotic cells, enzymatic degradation in the human body and low selectivity [13,14]. One of the reasons for our shortcomings is the still poor understanding of the relationship between peptide structure and its membrane activity, although dozens of models have been proposed [22,23,39,68].

Melittin and magainin have been known for decades as the typical examples of α-helical antimicrobial amphipathic peptides [13]. Therefore, plenty of studies have been devoted to elucidating the molecular mechanisms of their membrane activity. It is established that these peptides form so-called toroidal peptide–lipid pores when added to the lipid membranes at micromolar concentrations [22]. However, there are still some blind spots in the mechanisms of rearrangement of these peptides from the initial membrane-bound state, when the peptides are almost parallel to the membrane surface, to the transmembrane configuration of the pore. In general, antimicrobial peptides are predominantly amphipathic molecules. This means that their hydrophobic side is immersed in the membrane. One-sided peptide adsorption should therefore lead to an asymmetry in membrane tension. This asymmetry has been proposed to be the reason for the formation of membrane pores by melittin [67,69]. Therefore, the redistribution of the peptides between membrane monolayers due to their translocation through the transiently formed pores should diminish the tension asymmetry and lead to the inhibition of the pore formation process [67]. At the same time, some authors suggest the possibility of the peptide translocation without the formation of leaky pores for water-soluble fluorescent dyes [30]. Thus, translocation may not be accompanied by pore formation detectable by dye leakage. In addition, peptides can form purely lipidic pores [31] as well as facilitate lipid flip-flop [36]. Therefore, several types of pores can be formed by the peptides, depending on their concentration and membrane composition [13].

In [26,27,28], the authors propose a classification of the electrical signals induced by the antimicrobial peptides, starting from millisecond spike signals to square-top signals, whose duration is much longer. However, these structures are typically observed in the micromolar range of the bulk peptide concentration. But what about the early stages of peptide adsorption? Experiments with dye leakage from vesicles or patch-clamp measurements typically cannot provide any information about this stage. Our IFC experiments showed that peptides adsorb to the membrane at P/L lower than the pore-forming limit (70 nM for melittin and 80 nM for magainin) peptides adsorb onto the membrane (see Figure 4). At the same time, we did not observe any electrical activity for such peptide concentrations using patch-clamps (Figure 2a,b). In IFC experiments, the measured value of the boundary potential is a sum of the surface potential, which reflects the appearance of a charge at the membrane/water interface, and the dipole potential, which results from the redistribution of the lipid polar headgroups and associated water molecules upon addition of molecules (either charged or uncharged) to this region of the membrane. If the adsorbing peptide molecule remains predominantly superficial at the membrane interface, it should only affect the surface potential, *φ*(0). According to the GCS theory, the value of this potential is related to the surface charge density at the membrane interface:

(3) $$\sigma =\sqrt{8RT\varepsilon \varepsilon_{0}C_{el}}sinh\left(\frac{zF\varphi \left(0\right)}{2RT}\right)$$

Thus, for equal values of *σ*, *φ*(0) should be lower at high ionic strength (high value of *C_el_*) compared with low ionic strength conditions. For magainin, the amplitude of the Δφ_b_ after the first addition of 80 nM peptide was two times higher in the 10 mM KCl buffer compared with the 100 mM KCl buffer (Table 1). This means that this molecule was shallowly immersed in the membrane and mainly changed the surface potential term of φ_b_. However, the difference between 100 mM KCl and 10 mM KCl should result in a nearly threefold difference in the *φ*(0) value. Thus, magainin should also change the dipole potential by inserting its hydrophobic side into the lipid bilayer. In contrast, there was no difference between high and low ionic strength conditions for melittin (Table 1). This suggests that melittin penetrates deeper into the membrane and only affects the value of the dipole potential, which is insensitive to the ionic strength of the buffer solution. These results are consistent with MD simulations showing that magainin produces a greater stress on the bilayer compared with melittin [49].

After the second addition of the peptides, we reached the pore-forming concentrations of 140 nM melittin and 160 nM magainin, as follows from patch-clamp experiments (Figure 2c,d). We observed all types of the conducting events: spike, multi-level, and square-top signals. However, for melittin, the relative number of multi-level and square-top signals was lower than that of spike signals (Figure 3a), while for magainin, spike signals were the least abundant (Figure 3b). At the same time, the total number of conducting events was lower for magainin than for melittin. Under high ionic strength conditions, our IFC measurements showed a very small decrease in the Δφ_b_ for 140 nM melittin, while for 160 nM magainin the difference in the boundary potentials decreased to almost zero in about 10 min (Figure 4a,b, Table 1). It is noteworthy that the setup of the IFC method requires that the transmembrane potential be zero through the feedback loop. A negative transmembrane potential difference is thought to facilitate pore formation and translocation of positively charged peptide molecules, whereas a positive potential difference should have the opposite effect [34,38,39,40]. Thus, the IFC technique is free from possible artifacts resulting from the transmembrane potential difference. Therefore, our results suggest that short-living spike signals observed predominantly for melittin may not be related to peptide translocation but may reflect the formation of lipidic pores. Thus, we propose that the formation of a smaller number of toroidal pores relative to the number of lipidic pores (Figure 3a) results in very negligible translocation for melittin, whereas the formation of a large number of toroidal pores (Figure 3b) results in extensive translocation for magainin.

Indeed, the idea of a possible translocation of melittin is based on the results of FRET experiments with asymmetric addition of labeled lipids and peptides [34,35]. However, the appearance of the pure lipidic pore between two peptide molecules, as proposed in [31], should make a bridge between lipid monolayers, which would result in a uniform redistribution of labeled lipids between membrane leaflets. Thus, even in the case of a peptide-free lipidic pore, one could detect the change in the FRET signal. As it has been proposed in [70], a deeper insertion of the peptide into the lipid bilayer will result in a less stressed state of the membrane. As demonstrated by the analysis of the obtained results of the Δφ_b_ change for 70 nM melittin and 80 nM magainin at high and low ionic strength (Table 1), melittin inserts deeper into the lipid bilayer compared with the magainin. This results in the generation of a greater number of spike signals associated with lipidic pores for melittin than for magainin. The formation of these pores relieves the stress of the asymmetric membrane tension resulting from the one-sided adsorption of the peptides onto the membrane. Consequently, in the absence of asymmetry in membrane tension, extensive translocation of the peptides is no longer required. However, the presence of both multi-level and square-top signals (Figure 2c and Figure 3a) shows that melittin also forms toroidal pores. This is consistent with our results showing a slight decrease in the Δφ_b_ in the case of melittin (Figure 4a). Thus, we can propose that at these surface concentrations (P/L about 1/100), melittin tends to form predominantly small lipidic pores and a few toroidal pores, which contribute to the negligible translocation.

At the same time, some authors consider the possibility of the peptide translocation into vesicles without leakage of the water-soluble dye [30]. We also suspect that some smaller defects precede electrically detectable pores at very low peptide concentrations, beyond the detection limits of our methods. Under low ionic strength conditions, we did not observe any decrease in the boundary potential difference upon sequential addition of melittin (Figure 4c). Based on this observation, we can assume that the translocation of the peptides requires the preliminary formation of a membrane defect, which could not be formed at low ionic strength due to the strong electrostatic repulsion between the charged peptide molecules.

In contrast to melittin, our patch-clamp measurements suggest that magainin predominantly forms toroidal pores when the threshold concentration of P/L is exceeded by about 1/60. However, even these pores are transient structures that disappear with time, and the 10 min decrease in the Δφ_b_ (Figure 4b) is a result of random redistribution of magainin molecules between membrane leaflets, which could be described as peptide translocation (Table 1). Under low ionic strength conditions, we observed a very slight decrease in the Δφ_b_ after the second addition of the peptide (see Figure 4d), which further supports the idea of predominantly toroidal peptide–lipid pores for magainin; such pores facilitate peptide translocation.

**Figure 5:**
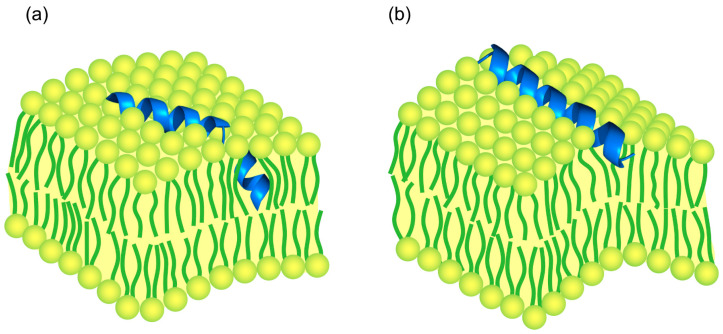
Model of peptide insertion into the lipid bilayer for (**a**) melittin and (**b**) magainin. The shallow insertion of magainin corresponds to the greater added lateral area and stress of the lipid packing compared with melittin.

In conclusion, our results provide experimental evidence for the molecular mechanism underlying the difference in pore formation by melittin and magainin, as well as qualitative insights into the molecular structure of the corresponding pores. In support of the idea of [31], we propose that antimicrobial peptides can form pure lipidic pores, while their translocation occurs predominantly through transient toroidal peptide–lipid pores. For a given surface concentration, the choice between pore types is determined by the depth of peptide insertion and the added lateral area occupied by the peptide in a membrane leaflet. The greater the area of peptide insertion, the greater the stress asymmetry imposed on the membrane. Melittin consists of a longer peptide chain with a proline kink dividing two helices, whereas magainin is shorter and consists of a single helix. Thus, we propose that the more flexible structure of melittin with a proline kink, which allows the peptide to adopt specific conformational states in the membrane, results in deeper peptide insertion into the lipid bilayer and a less stressed state of the lipid bilayer (Figure 5a). Consequently, melittin tends to form a greater number of lipidic pores, causing only negligible peptide translocation across the membrane. In contrast, magainin forms predominantly toroidal pores, promoting extensive peptide translocation because its molecule adds a greater lateral area to the membrane, significantly disrupting lipid packing (Figure 5b). Thus, our results suggest a new rationale for the structure–function relationship of antimicrobial peptides.

## Figures and Tables

**Table 1:** The change in the Δφ_b_ after each addition for melittin and magainin in the buffer solutions with low and high ionic strength.

Melittin			Magainin		
Concentration, nM	Δφ_b_ Change at Low Ionic Strength, mV	Δφ_b_ Change at High Ionic Strength, mV	Concentration, nM	Δφ_b_ Change at Low Ionic Strength, mV	Δφ_b_ Change at High Ionic Strength, mV
70	9 ± 2	10 ± 3	80	16 ± 3	8 ± 3
140	5 ± 2	8 ± 3	160	11 ± 2	7 ± 2
Characteristic time of the Δφ_b_ decrease, min	-	70 ± 7		-	9 ± 2

## Data Availability

All datasets are available on demand.
